# A Measure of the Broad Substrate Specificity of Enzymes Based on ‘Duplicate’ Catalytic Residues

**DOI:** 10.1371/journal.pone.0049313

**Published:** 2012-11-16

**Authors:** Sandeep Chakraborty, Bjarni Ásgeirsson, Basuthkar J. Rao

**Affiliations:** 1 Department of Biological Sciences, Tata Institute of Fundamental Research, Mumbai, India; 2 Department of Biochemistry, Science Institute, University of Iceland, Reykjavik, Iceland; 3 Department of Biological Sciences, Tata Institute of Fundamental Research, Mumbai, India; UMR-S665, INSERM, Université Paris Diderot, INTS, France

## Abstract

The ability of an enzyme to select and act upon a specific class of compounds with unerring precision and efficiency is an essential feature of life. Simultaneously, these enzymes often catalyze the reaction of a range of similar substrates of the same class, and also have promiscuous activities on unrelated substrates. Previously, we have established a methodology to quantify promiscuous activities in a wide range of proteins. In the current work, we quantitatively characterize the active site for the ability to catalyze distinct, yet related, substrates (BRASS). A protein with known structure and active site residues provides the framework for computing ‘duplicate’ residues, each of which results in slightly modified replicas of the active site scaffold. Such spatial congruence is supplemented by Finite difference Poisson Boltzmann analysis which filters out electrostatically unfavorable configurations. The congruent configurations are used to compute an index (BrassIndex), which reflects the broad substrate profile of the active site. We identify an acetylhydrolase and a methyltransferase as having the lowest and highest BrassIndex, respectively, from a set of non-homologous proteins extracted from the Catalytic Site Atlas. The acetylhydrolase, a regulatory enzyme, is known to be highly specific for platelet-activating factor. In the methyltransferase (PDB: 1QAM), various combinations of glycine (Gly38/40/42), asparagine (Asn101/11) and glutamic acid (Glu59/36) residues having similar spatial and electrostatic profiles with the specified scaffold (Gly38, Asn101 and Glu59) exemplifies the broad substrate profile such an active site may provide. ‘Duplicate’ residues identified by relaxing the spatial and/or electrostatic constraints can be the target of directed evolution methodologies, like saturation mutagenesis, for modulating the substrate specificity of proteins.

## Introduction

The remarkable ability of enzymes to selectively catalyze the reactions of compounds from the cellular soup is essential for the proper functioning of most pathways in biological systems [1], [2]. Simultaneously, evolution has endowed these enzymes with flexibility and plasticity to catalyze the conversion of a wide range of related substrates [3]–[5]. In certain cases, such broad substrate specificity poses serious concerns, as in the emergence of extended-spectrum β-lactamases generating multiresistant strains of bacteria [6], [7]. The structural and molecular basis of broad substrate specificity has been the subject of intense research in diverse fields like drug design, industrial applications, etc. [8]–[12]. Substrate transition state stabilization is another trait that has been selected for during evolution, since it ensures high catalytic efficiency [13].

A quantitative measure of broad substrate specificity is yet to be formalized. A previous attempt to quantify broad substrate specificity provided a measure of the catalytic efficiencies of an enzyme toward a pre-defined set of substrates, but was limited in its scope and scalability [14]. A trait related to broad substrate specificity is promiscuity, which is defined as the catalysis of reactions distinct from the one the protein has evolved to perform, but using the same active site scaffold [15]–[18]. Previous work by our group has established a methodology to quantify promiscuous activities in a wide range of proteins [19], [20].

In the current work, we quantitatively characterize the active site of an enzyme to measure broad substrate specificity - **Br**o**a**d **s**ubstrate **s**pecificity estimator (BRASS). BRASS computes ‘duplicate’ residues from the structure of a protein with known active site residues. These ‘duplicate’ residues generate slightly modified replicas of the active site scaffold. Finite difference Poisson Boltzmann analysis is used to filter out electrostatically unfavorable configurations [21], [22]. We compute an index (BrassIndex) by the number of configurations that are equivalent to the active site scaffold, such that at least one residue in each new configuration is present in the original active site motif. Thus, we ignore any possible moonlighting functions, where the main active site would not be the host to the catalytic residues [23].

We identified an acetylhydrolase and a methyltransferase as having the lowest and highest BrassIndex, respectively, from a set of proteins extracted from the Catalytic Site Atlas [24]. Moreover, there was no correlation found between the BrassIndex and the promiscuity index (as computed in [20]) of a protein. BRASS analysis can be easily adapted to directed evolution methodologies. The target residues can be identified by relaxing the spatial and/or electrostatic constraints in the current flow, and these ‘**duplicate**’ residues can be subjected to saturation mutagenesis which selects for high specificity with respect to a desired substrate [13]. To summarize, we present a methodology for detecting congruent scaffolds in the active site of a protein, which we hypothesize results in broad substrate specificity. We quantitatively characterize these properties in a wide range of proteins.

## Results

Table 1 shows the proteins with the highest and lowest degree of substrate specificity as defined by the index (BrassIndex) computed by BRASS. BRASS identified an acetylhydrolase as a protein with the lowest BrassIndex, and a methyltransferase and a thioesterase as proteins possessing the highest BrassIndex in the set of 420 proteins under consideration. We now discuss each of these three proteins with respect to their active sites.

**Table 1:** Proteins with highest and lowest BrassIndex : L = sequence length.

	PDB	L	Description
	1QAM	244	ERMC' METHYLTRANSFERASE
	1OH9	258	ACETYLGLUTAMATE KINASE
	1EH5	279	PALMITOYL THIOESTERASE 1
	1QGX	357	3'-5'-ADENOSINE BISPHOSPHATASE
Highest	1DUB	261	2-ENOYL-COA HYDRATASE
	2JXR	329	PROTEINASE A
	2HGS	474	GLUTATHIONE SYNTHETASE
	1D8H	311	mRNA TRIPHOSPHATASE CET1
	1D2H	292	GLYCINE N-METHYLTRANSFERASE
	1HZD	272	AU-BINDING PROTEIN/ENOYL-COA HYDRATASE
	1BWP	233	PLATELET-ACTIVATING FACTOR ACETYLHYDROLASE
	1AKO	268	EXONUCLEASE III
	1L1L	739	RIBONUCLEOSIDE TRIPHOSPHATE REDUCTASE
	2DLN	306	D-ALANINE–D-ALANINE LIGASE
	1NWW	149	LIMONENE-12-EPOXIDE HYDROLASE
Lowest	1EI5	520	D-AMINOPEPTIDASE
	1NBA	264	N-CARBAMOYLSARCOSINE AMIDOHYDROLASE
	1EC9	446	GLUCARATE DEHYDRATASE
	3R1R	761	RIBONUCLEOTIDE REDUCTASE R1
	1STD	172	SCYTALONE DEHYDRATASE

### 1. Highest BrassIndex: rRNA Methyltransferase (PDBid:1QAM)

The critical role of the ribosome as the site for protein synthesis in cell viability makes it the logical target for a wide range of drugs [25]. In response to the drug challenge, pathogens develop resistance through mutations and methylations [26]. Methyltransferases (MTases) transfer a methyl group from a donor to an acceptor, and catalyze a diverse range of substrates (small organic molecules, DNA, RNA, proteins, lipids) [27]. The rRNA MTase ErmC' methylates an adenine base in 23S rRNA, which confers resistance by obstructing the contact site for the antibiotics [28].

**Figure 1:**
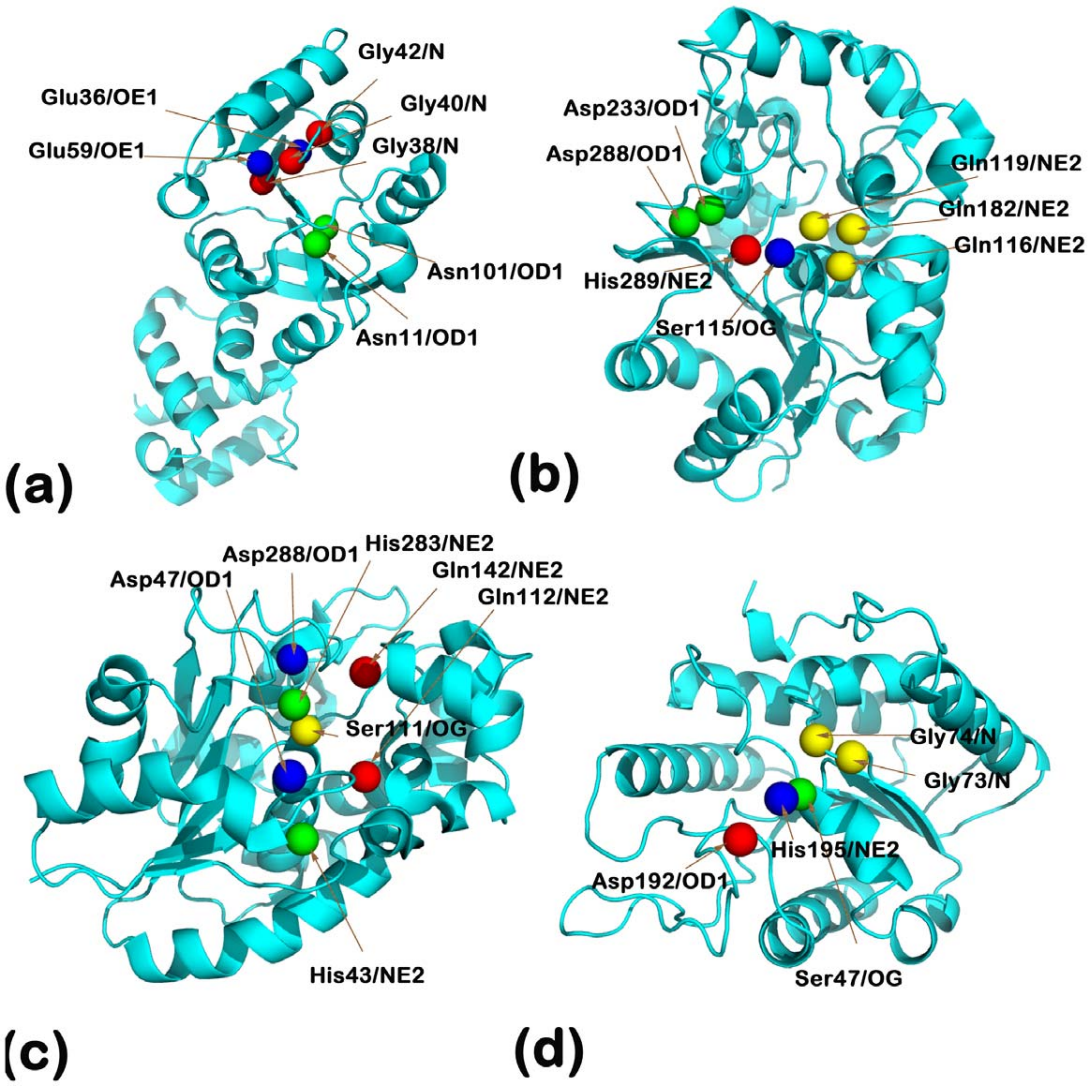
Active sites of proteins with the highest and lowest BrassIndex. (**a**) rRNA Methyltransferase (PDBid:1QAM): This protein has the highest BrassIndex, as can be seen by the presence of various similar residues in close proximity, that results in electrostatically similar scaffolds as well (Table 2). (**b**) Palmitoyl protein thioesterase 1 (PPT1) (PDBid:1EH5): Protein with the next highest BrassIndex. We hypothesize that a ‘replica’ catalytic triad consisting of Asp288 exists and is congruent to the known catalytic triad (His289, Asp233, Ser115) (Table 3). (**c**) Palmitoyl protein thioesterase 2 (PPT2) (PDBid:1PJA): PPT2 has a 26% similarity with PPT1, but has a non-redundant role in the cell. The absence of supporting residues could be a possible reason why PPT2 is unable to act upon all compounds (particularly those with have bulky head groups) which PPT1 catalyzes. (**d**) Platelet-activating acetylhydrolase (PDBid:1BWP): This protein has the lowest BrassIndex, which is due to the absence of ‘duplicate’ residues in the proximity of the core active site residues (Table 5). This implies that this protein has high specificity, a fact that has been noted in [39].

Fig. 1a shows the site in the rRNA MTase ErmC' (PDBid:1QAM) that binds the cofactor S-adenosyl-L-methionine (AdoMet). It can be seen that the residues Gly/38/40/42, Asn101/11 and Glu59/36 present possible combinations that are spatially and electrostatically congruent (Table 2). It has been noted that ``in the AdoMet complex, the positively charged sulfur atom of the methionine moiety interacts with main-chain carbonyl groups of Asn11 and Asn101" [28]. Furthermore, in the proposed transition state of transmethylation catalyzed by ErmC', a “favorable region for accommodating the N6 of adenine” has been observed due to contact with the O atom of Asn101 and Asn11 [28]. Thus, the active site can be seen to both stabilize the cofactor, as well as provide a binding site for a broad profile of recognized substrates. The broad substrate profile of the enzyme is amply demonstrated by its ability to methylate increasingly truncated nucleotide transcripts of the domain methylated in 23S rRNA, down to a minimal of a 27 nucleotide long stem-loop sequence [29].

**Table 2:** Potential and spatial congruence of ‘duplicate’ scaffolds to the active site in a methyltransferase (PDBid:1QAM).

Active site atoms (a,b,c)		ab	ac	bc
GLY38N,ASN101OD1,GLU59OE1,	D	9.5	4.7	10.9
	PD	157.8	153.8	−4
GLY40N,ASN101OD1,GLU59OE1,	D	8.8	3.7	10.9
	PD	222.6	218.6	−4
GLY38N,ASN101OD1,GLU36OE1,	D	9.5	5.9	10.3
	PD	157.8	234.8	77.1
GLY40N,ASN101OD1,GLU36OE1,	D	8.8	5.9	10.3
	PD	222.6	299.6	77.1
GLY42N,ASN101OD1,GLU36OE1,	D	11	6.2	10.3
	PD	218.4	295.5	77.1
GLY40N,ASN11OD1,GLU59OE1,	D	11.8	3.7	12.9
	PD	184.4	218.6	34.2

The active site residues specified are Gly38, Asn101 and Glu59. D = Pairwise distance in Å. PD = Pairwise potential difference. See Methods section for units of potential.

### 2. High BrassIndex: Palmitoyl-protein Thioesterase (PDBid:1EH5)

Palmitoylation is a critical protein modification that governs protein-protein interactions, protein trafficking and membrane localization [30], [31]. Depalmitoylation is known to be carried out either by lysosomic or cytoplasmic thioesterases [32]. Palmitoyl-protein thioesterase 1 (PPT1) is a lysosomal enzyme that is responsible for the removal of fatty acyl groups from cysteine residues in modified proteins [33]. Mutations in the gene that encodes PPT1 have been ascertained as the primary cause of neuronal ceroid lipofuscinoses, a family of recessively inherited childhood neurodegenerative disorders. In the PPT1 protein (PDBid: 1EH5), multiple glutamines (Gln116/119/182) are spatially and electrostatically equivalent to the catalytic triad (His289, Asp233 and Ser115) (Fig. 1b and Table 3). Interestingly, the Asp233 has an equivalent Asp288 in its close vicinity, which might constitute another triad for proteolytic purposes. This hypothesis is apparently refuted by the fact that the D233N mutant resulted in null activity. However, this loss in activity might be due to an improperly folded protein - as is believed to have happened for the triple mutant Asn(197/212/232)Gln by Bellizzi et al. [33]. The Asp288 triad theory garners more support from the fact that the identity of the third member of the catalytic triad could not be ascertained before the completion of the structure determination, as several candidates were suggested by the mutagenesis data, and that “Asp233 was a surprising finding” [33].

**Table 3:** Potential and spatial congruence of ‘duplicate’ scaffolds to the active site in palmitoyl-protein thioesterase 1 (PDBid:1EH5).

Active site atoms (a,b,c,d)		ab	ac	ad	bc	bd	cd
GLN116NE2,HIS289NE2,ASP233OD1,SER115OG,	D	9.7	14.2	6.3	5.3	3.6	8.3
	PD	73.4	111.6	−60.5	38.3	−133.9	−172.1
GLN182NE2,HIS289NE2,ASP233OD1,SER115OG,	D	10.9	14.2	8.1	5.3	3.6	8.3
	PD	64.2	102.5	−69.6	38.3	−133.9	−172.1
GLN116NE2,HIS289NE2,ASP288OD1,SER115OG,	D	9.7	16.5	6.3	7.4	3.6	10.8
	PD	73.4	27.3	−60.5	−46	−133.9	−87.8
GLN119NE2,HIS289NE2,ASP288OD1,SER115OG,	D	9.1	15.6	6.2	7.4	3.6	10.8
	PD	177.1	131	43.2	−46	−133.9	−87.8
GLN182NE2,HIS289NE2,ASP288OD1,SER115OG,	D	10.9	16.5	8.1	7.4	3.6	10.8
	PD	64.2	18.2	−69.6	−46	−133.9	−87.8

The active site residues specified are Gln116, His289, Asp233, Ser115. D = Pairwise distance in Å. PD = Pairwise potential difference. See Methods section for units of potential.

Moreover, PPT1 is known to have a much broader substrate profile than a related thioesterase (PPT2), with which it shares 26% identity [32], [34]. This corroborates the high BrassIndex computed for PPT2 using BRASS. Firstly, BRASS is able to extract the correct catalytic residues in PPT2 (Gln112, His283, Asp228 and Ser111) using the active site motif (Gln116, His289, Asp233 and Ser115) from PPT1 (Table 4). However, there are no ‘duplicate’ residues in the vicinity for PPT2 (Fig. 1c). It can be seen that the best match with the active site scaffold has a distance of 7.5 Å between Gln142 and His283, in place of a distance of 13.3 Å in the cognate pair of Gln112 and His283 (Table 4). Such a constricted site, with respect to PPT1, may also explain the lack of activity of PPT2 against substrates that have bulky head groups [34].

**Table 4:** Potential and spatial congruence of ‘duplicate’ scaffolds to the active site in palmitoyl-protein thioesterase 2 (PDBid:1PJA).

Active site atoms (a,b,c,d)		ab	ac	ad	bc	bd	cd
GLN112NE2,HIS283NE2,ASP228OD1,SER111OG,	D	9.9	13.3	7.3	4.6	2.8	6.9
	PD	−72.3	22.9	−146.3	95.2	−74.1	−169.3
GLN142NE2,HIS283NE2,ASP228OD1,SER111OG,	D	8.4	7.5	8.5	4.6	2.8	6.9
	PD	−50.3	44.9	−124.4	95.2	−74.1	−169.3
GLN142NE2,HIS283NE2,ASP47OD1,SER111OG,	D	8.4	16.7	8.5	10.3	2.8	9.8
	PD	−50.3	39.2	−124.4	89.5	−74.1	−163.6
GLN112NE2,HIS43NE2,ASP47OD1,SER79OG,	D	10	12.3	6.2	7.4	6.6	8.4
	PD	43.3	17.3	−131.9	−26	−175.2	−149.2

The active site residues specified are Gln112, His283, Asp228, Ser111. D = Pairwise distance in Å. PD = Pairwise potential difference. See Methods section for units of potential.

### 3. Lowest BrassIndex: Platelet-activating Factor Acetylhydrolase (PDBid:1BWP)

Platelet-activating factor (PAF) is a very potent messenger phospholipid, found in picomolar concentrations in fluids like cytosol, blood plasma and urine [35]. PAF is implicated in several critical physiological pathways, like activation of platelets and monocytes [36] and modulation of cell proliferation [37]. The levels of PAF are tightly regulated by PAF acetylhydrolases (PAF-AH), which inactivate PAF by hydrolyzing the ester bond [38]. Fig. 1d shows the active site residues of the alpha-1 subunit of the isoform Ib of bovine brain intracellular PAF-AH, which indicates very few ‘duplicate’ residues [39]. This fact is corroborated by data shown in Table 5, which shows only one additional scaffold that has both spatial and electrostatic congruence with the specified catalytic motif. Y.S. Ho et al. have emphasized this very high specificity (``highly specific for PAF and effectively do not hydrolyze phospholipids with acyl chains longer than acetate in the sn-2 position"), noting that this feature is “a hallmark of a regulatory hydrolase” [39]. The ability of the BRASS methodology to specifically select out such a protein as being highly selective adds confidence to its underlying principle.

**Table 5:** Potential and spatial congruence of ‘duplicate’ scaffolds to the active site in a acetylhydrolase (PDBid:1BWP).

Active site atoms (a,b,c,d)		ab	ac	ad	bc	bd	cd
ASP192OD1,SER47OG,HIS195NE2,GLY74N,	D	8.1	5.4	11.9	3.2	5.2	6.5
	PD	−128.5	29.5	−177.2	157.9	−48.7	−206.6
ASP192OD1,SER47OG,HIS195NE2,GLY73N,	D	8.1	5.4	13.1	3.2	5.8	7.8
	PD	−128.5	29.5	−188.4	157.9	−60	−217.9
ASP75OD1,SER76OG,HIS79NE2,GLY102N,	D	7.1	5.9	9.8	5.1	6.8	10.9
	PD	−146.9	−144.3	−239.8	2.5	−93	−95.5
ASP46OD1,SER47OG,HIS195NE2,GLY74N,	D	4.3	7.1	6.3	3.2	5.2	6.5
	PD	−139.2	18.7	−187.9	157.9	−48.7	−206.6
ASP182OD1,SER186OG,HIS180NE2,GLY177N,	D	9.3	6.1	12.5	4.7	11.5	8.7
	PD	−121.9	4.4	−218.6	126.4	−96.6	−223

The active site residues specified are Asp192, Ser47, His195, Gly74.

D = Pairwise distance in Å. PD = Pairwise potential difference. See Methods section for units of potential.

### 4. Relaxing Spatial Constraints and Allowing Stereochemical Equivalence

CLASP provides the flexibility in the search process by allowing a user to provide a defined set of residues to match a particular position of the active site motif. Furthermore, the search algorithm is parameterized to exclude matches if any pairwise distance deviation exceeds a user specified threshold. In the default mode, CLASP scores any pairwise deviation more than 2 Å in distance highly, thus eliminating that match as a significant one. We relaxed the spatial constraint, increasing this threshold distance to 5 Å. We created the following groups - BASIC = [Lys His Arg], ACIDIC = [Glu Asp], AMIDE = [Asn Gln], NONPOLAR = [Gly Ala Val Leu Ile Met], AROMATIC = [Phe Trp Tyr], and applied the BRASS algorithm to the chosen set of proteins under consideration.

The more flexible search by BRASS identified a ketosteroid isomerase (KSI) (PDBid:1C7H), which catalyzes an allylic isomerization reaction at a diffusion-controlled rate, with a high BrassIndex. The KSI uses a network of hydrogen bonds to connect the critical residues Tyr16 and Asp103. Apart from these two critical residues, Asp40 and Val104 constituted the active site motif as specified in the CSA database (Asp103, Tyr16, Asp40 and Val104). The non-critical residues were allowed to be substituted by stereochemically equivalent residues, i.e. Asp104 and Val104 could be replaced by the elements of the groups BASIC and NONPOLAR respectively. Table 6 shows that the vicinity of Val104 has the following non-polar residues that may take up an equivalent role - [Ile102, Ala118, Val101, Met105, Met116, Met84, Val104, Val88, Val38, Ala118, Ile17, Ala83], a finding that aligns well with the highly non-polar nature of the active site. Furthermore, although the distance deviations are high, Glu39 can be seen to be a possible substitute for Asp40.

**Table 6:** Potential and spatial congruence of ‘duplicate’ scaffolds to the active site in a ketosteroid isomerase (PDBid:1C7H).

Active site atoms(a,b,c,d)		ab	ac	ad	bc	bd	cd
ASP103OD1,TYR16OH,ASP40OD1,VAL104N,	D	5.8	6.6	4.8	8.5	8.8	10
	PD	−99.5	−20.7	−284.8	78.8	−185.3	−264.2
ASP103OD1,TYR16OH,ASP40OD1,ILE102N,	D	5.8	6.6	4.6	8.5	10.1	8.4
	PD	−99.5	−20.7	−192.6	78.8	−93.1	−171.9
ASP103OD1,TYR16OH,ASP40OD1,ALA118N,	D	5.8	6.6	4.6	8.5	8.5	5.5
	PD	−99.5	−20.7	−214	78.8	−114.5	−193.3
ASP103OD1,TYR16OH,ASP40OD1,VAL101N,	D	5.8	6.6	6.6	8.5	10.9	8.9
	PD	−99.5	−20.7	−247.6	78.8	−148.1	−226.9
ASP103OD1,TYR16OH,ASP40OD1,MET105N,	D	5.8	6.6	7.3	8.5	9.3	12.3
	PD	−99.5	−20.7	−313.8	78.8	−214.3	−293.1
ASP103OD1,TYR16OH,ASP40OD1,MET116N,	D	5.8	6.6	8.5	8.5	8.5	10.5
	PD	−99.5	−20.7	−323.2	78.8	−223.8	−302.6
ASP103OD1,TYR16OH,ASP40OD1,MET84N,	D	5.8	6.6	7	8.5	10.7	13.3
	PD	−99.5	−20.7	−263.8	78.8	−164.3	−243.2
ASP103OD1,TYR16OH,GLU39OE1,VAL104N,	D	5.8	11.4	4.8	13	8.8	9.9
	PD	−99.5	−103.9	−284.8	−4.4	−185.3	−180.9
ASP103OD1,TYR16OH,ASP40OD1,VAL88N,	D	5.8	6.6	9.6	8.5	11.1	11.1
	PD	−99.5	−20.7	−266.8	78.8	−167.3	−246.2
ASP103OD1,TYR16OH,ASP40OD1,VAL38N,	D	5.8	6.6	10.8	8.5	9.8	9.6
	PD	−99.5	−20.7	−237.5	78.8	−138	−216.9
ASP103OD1,TYR16OH,GLU39OE1,ALA118N,	D	5.8	11.4	4.6	13	8.5	7.3
	PD	−99.5	−103.9	−214	−4.4	−114.5	−110.1
ASP103OD1,TYR16OH,ASP40OD1,ILE17N,	D	5.8	6.6	9.5	8.5	6.8	14.3
	PD	−99.5	−20.7	−325.9	78.8	−226.4	−305.2
ASP103OD1,TYR16OH,ASP40OD1,ALA83N,	D	5.8	6.6	9.2	8.5	12.2	15.3
	PD	−99.5	−20.7	−325.4	78.8	−225.9	−304.7

The active site residues specified are Asp103, Tyr16, Asp40, Val104. D = Pairwise distance in Å. PD = Pairwise potential difference. See Methods section for units of potential.

### 5. Alkaline Phosphatase

Zinc binding sites are classified as catalytic, structural or cocatalytic [40]. In cocatalytic sites, a single amino acid residue (usually Asp or Glu) ligands two zinc ions in addition to other ligands as the zinc ions are often penta-coordinate and arranged in a trigonal bipyramidal geometry. In a cold-active *Vibrio* alkaline phosphatase (VAP) (PDBid: 3E2D), there are three metal ions similar to most other alkaline phosphatases - the M1 site (Asp273, His277, His465) binds a zinc ion, the M2 site (Ser65, Asp315, His316, Asp12) binds another zinc ion, while the M3 site (Asp12, His116, Thr118, Glu268) binds a magnesium ion [41]. Asp12, which is observed to ligand both the M2 and M3 ions, can be seen to be redundant with Asp315 filling in for it (Table 7). It must be admitted that the congruence is not such that it inspires absolute confidence, and mutational studies would be required to confirm this hypothesis. APs are broad specificity enzymes due to the fact that the phosphorylgroup is the main chemical group of the substrate that enters the active site and makes bonds with the enzyme. Thus, the positional variants for the substrate are limited.

**Table 7:** Residues liganding magnesium ion in VAP.

Active site atoms (a,b,c,d)		ab	ac	ad	bc	bd	cd
ASP12OD1,HIS116NE2,THR118OG1,GLU268OE1,	D	8.2	5.1	7	7.2	7.3	3.8
	PD	−460.7	−206.5	−291.6	254.2	169	−85.2
ASP315OD1,HIS116NE2,THR118OG1,GLU268OE1,	D	10.9	5.8	8.3	7.2	7.3	3.8
	PD	−282.3	−28.1	−113.3	254.2	169	−85.2

Asp315 can be seen to able to partially substitute for Asp12. D = Pairwise distance in Å. PD = Pairwise potential difference. See Methods section for units of potential.

### 6. Frequency Distribution and Comparison to the Promiscuity Index

**Figure 2:**
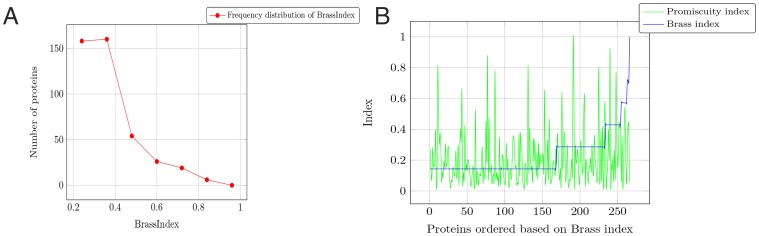
Statistics of BrassIndex on the population: (a) Frequency distribution of BrassIndex. It can be seen that most proteins are highly specific (low BrassIndex), and the number of proteins with high specificity drops exponentially. **(b) Lack of** **correlation between promiscuity and substrate specificity (Brass) indices**: As expected, there is no correlation between promiscuity (defined as the ability to catalyze reactions distinct from the one the protein has evolved to perform, but using the same active site scaffold) and the ability of enzymes to catalyze the reaction of different, but related, compounds using the same catalytic mechanism (broad substrate specificity). The promiscuity indices are computed as described in [20].

Fig. 2a shows the frequency distribution of the BrassIndex on the set of 420 proteins. It is seen that most proteins have a low BrassIndex (high specificity). The number of proteins with broad specificity (high BrassIndex) drops almost exponentially. We have previously established a methodology to quantify promiscuous activities in a wide range of proteins [20]. There was no detectable correlation between the promiscuity index (computed as described in [20]) and the BrassIndex (Fig. 2b). Furthermore, BrassIndex was found to be independent of the threshold CLASP score used to prune out unfeasible scaffolds.

## Discussion

Evolution has shaped enzymes in accordance to their niche in the cell. Varied physiological constraints have molded enzymes to be mostly efficient [42], at times highly promiscuous [43], often precisely selective [39] and generally to possess broad, but related substrate profiles [11]. Often, it is untenable to provide an *in vitro* measure of these characteristics due to the laborious work involved, and the huge number of enzymes being discovered [14]. The rapid advancement in technology has allowed *in silico* methodologies to address this requirement with predictive models for some of these characteristics [20].

In the current work, we quantitatively characterize the active site of an enzyme to measure broad substrate specificity - **Br**o**a**d **s**ubstrate **s**pecificity estimator (BRASS). We hypothesize that ‘duplicate’ residues generate slightly modified, both spatially and electrostatically, replicas of the active site scaffold that are responsible for a broad substrate profile. Finite difference Poisson Boltzmann analysis is used as a discriminator to rule out electrostatically unfavorable configurations [21], [22]. BRASS computes an index (BrassIndex) using the number of configurations that are equivalent to the active site scaffold. Furthermore, permutations of the original active site residues are also excluded, due to the inability of our method to distinguish between mirror images.

BRASS was applied to a set of non-homologous proteins extracted from the Catalytic Site Atlas [24]. The frequency distribution curve suggested that most proteins have high specificity (Fig. 2a). This probably explains why promiscuity and broad specificity are relatively new concepts [15], usurping the older hypothesis that proteins are highly specific [44]. As expected, there was no correlation found between the BrassIndex and the promiscuity index (as computed in [20]) of a protein (Fig. 2b). The residues, which are responsible for the promiscuous functionality, in the vicinity of the catalytic residues may or may not bestow additional specificity to the primary catalytic activity. This uncertainty results in the lack of correlation in the promiscuity and broad specificity indices. A platelet-activating factor acetylhydrolase (PAF-AH) (PDBid:1BWP) was identified as a protein with the lowest BrassIndex in this set [39]. This PAF-AH, like most regulatory enyzmes, is known to be highly specific for PAF [38]. Alongside this high specificity (i.e. low BrassIndex), the PAF-AH has a low promiscuity index (0.11) [20]. These characteristics of regulatory enzymes ensure that they are not preoccupied in catalyzing irrelevant substrates or inhibited by them. The ability of BRASS to select out such a protein gives credence to its underlying theory. It is to be noted that this is an exceptional situation. As dictated by the requirements of a regulatory enzyme, it should have high specificity (low BrassIndex) and low promiscuity. A random enzyme with high specificity need not satisfy this low promiscuity criteria. Among the proteins with high BrassIndex was an rRNA methyltransferase (MTase) [28] and a palmitoyl-protein thioesterase (PPT1) [33]. Both these proteins are known to have broad substrate specificities. Furthermore, the substrate specificity of PPT1 is known to be broader than a homolog (PPT2), with which it shares 26% identity [34]. BRASS analysis corroborated this fact, since it failed to detect duplicate residues in PPT2 similar to the ones found in PPT1.

Thus, BrassIndex applies to a particular enzyme, and is not a characteristic of the class of enzymatic reactions. Directed evolution is a generic term for methods that mimic and accelerate evolution [45]–[48], often targeting the residues in the vicinity of the catalytic site to yield faster result [49]–[51]. These directed evolution methodologies can be applied to modulate specificities of a given enzyme based on the BRASS specified duplicate residues. The target residues can be identified by relaxing the spatial and/or electrostatic constraints. In the current flow, such electrostatic potential difference constraints prune out configurations that have the required spatial attributes, but are not electrostatically favorable [19]. However, the current method is not intended for rational design of new functions. Such methods bestow a non-existing function in a target protein, either by selecting a pre-existing scaffold [52]–[57], or by using *de novo* approaches [58]–[62].

The mechanisms underlying the broad specificities possessed by some enzymes have been the subject of intense research. Molecular dynamics simulations have been used to study the basis of the broad substrate profile of cytochrome P450 (CYP) 3A4, which catalyzes the oxidative degradation of a wide range of compounds [63]. The study concluded that `the broad substrate specificity of CYP3A4 stems from the malleability of a loop (residues 211–218) that resides in the vicinity of the channel connecting the active site and bulk solvent'. Table S1 shows that keeping the core catalytic residues (Thr309, Glu308, Phe435 and Cys442) constant, there are four possibilities for the torsional position of Phe215, a residue that has the maximum `change in dynamic flexibilities of the different regions of protein structure due to ligand binding'. Two of the four candidates lie in the loop 211–218. Another computational study evaluated the electrostatic and van der Waals interactions between substrates and active site residues in order to ‘provide a basis to understand the catalytic role of conserved residues, the substrate specificity, and the relative activity of favorable substrates’ [64]. Once again they concluded that `structural features of the substrate-binding site and the van der Waals and electrostatic interactions between substrates and the conserved residues lead to the broad substrate recognition'. It has been our attempt to quantify these parameters (using a static model) by exploiting the electrostatic and spatial congruence of neighboring residues.

The physiological needs that modulate the specificities of enzymes might be understood by a study of the evolutionary related penicillin binding proteins (PBP) and serine β-lactamases, where a nucleophilic serine in the conserved SXXK motif forms an acyl-complex with β-lactam antibiotics [65]. Ideally, PBP's should have evolved to be very specific for the D-alanyl-D-alanine end of the peptidoglycan, which they cross-link as the last step of peptidoglycan synthesis. The β-lactam antibiotics mimic the D-alanyl-D-alanine portion and act as ‘suicide substrates’, thus inhibiting cell wall synthesis. The broad specificity in PBP's is exploited by designing β-lactam drugs (penicillins, carbapenems and cephalosporins) that conserve the β-lactam ring, but differ in other regions of their chemical structure. It has been hypothesized that β-lactamases have evolved from the PBP's to gain the ability to cleave the β-lactam ring, rendering the drugs ineffective [66]. The same phenomenon of broad specificity allows a single β-lactamase enzyme to hydrolyze a diverse range of drugs [7]. Conversely by reducing the range of the specificity of PBP's, the methicillin-resistant strains of *Staphylococcus aureus* have evolved a PBP (PBP2a) with low affinity for β-lactams [67].

BRASS essentially works on a static model. However, it implicitly includes dynamics based on the ‘flexibility’ of the main-chain scaffold and mobility of selected side chains inside prearranged folds or ensembles of conformation. The reason for ‘good’ binding or improved selectivity may be distal to the prime residues in the active site. These residues are considered by allowing for spatial variability, of course under the assumption that the distant residues primarily reinforce what their closer counterparts do by allowing for spatial variability. In alkaline phosphatases, residues in the secondary valence shell around the metal ions have effect on catalysis, e.q. by selecting magnesium in the M3-site and keeping correct coordination for catalysis (octahedral, tetrahedral, etc.) [68].

A caveat in the computation of an index like the BrassIndex is the reliability of the manual step in specifying the set of residues involved in the catalytic reaction. It is not possible to computationally figure out whether two closely placed residues are both essential or redundant for catalysis. In spite of this inevitable source of error, the number of residues that have proximal ‘duplicate’ candidates is a good benchmark for estimating BrassIndex. Electrostatic analysis of the congruence of the specified active site motif with the other ‘replicas’ is another metric used as a discriminator. It should be mentioned that BRASS is unable to detect if and how an enzyme has a specific stereospecificity or steroselectivity, and is simply guided by the specified active site residues. In summary, we quantitatively characterize the properties of an enzyme which results in broad substrate specificity, using spatial and electrostatic properties of residues in the active site and its close vicinity.

## Methods

### 1 Materials

Adaptive Poisson-Boltzmann Solver (APBS) and PDB2PQR packages were used to calculate the potential difference between the reactive atoms of the corresponding proteins [22], [69]. The APBS parameters were set as described previously in [19]. APBS writes out the electrostatic potential in dimensionless units of kT/e where k is Boltzmann's constant, T is the temperature in K and e is the charge of an electron. We extensively integrated and used the freely available BioPerl [70] modules and Emboss [71] tools. All protein structures were rendered by PyMol (http://www.pymol.org/).

### 2 Methodology

The underlying theoretical foundation for CLASP is the non-triviality of the spatial and electrostatic congruence in cognate pairs seen across various structures with the same catalytic mechanism. Table S2 shows the congruence in serine proteases. The two major families of serine proteases, chymotrypsin and subtilisin, are a classical example of convergent evolution where the catalytic Ser-His-Asp triad shows virtually similar geometry in the structurally different chymotrypsin and subtilisin [72]. This invariance in the electrostatic features (measured in structures that have been solved independently over many years) is an innate property required for the enzymatic activity. This also speaks highly of the reliability of the APBS/PDB2PQR implementation.

BRASS starts with a motif consisting of N residues from the catalytic site of a protein (P_i_) (Equation 1). All sets of N residues (Equation 2 and 3) are obtained in the same protein using an exhaustive search procedure similar to the one used in SPASM [73]. The pairwise distances and potential differences are computed for each match, and compared with the original motif. This generates the CLASP score, which defines an ordering of the matches (Equation 3). The CLASP score consolidates the distance and the potential difference scores, and reflects the likelihood that the activity in the reference protein exists in the query protein.

M_1_ represents the specified active site scaffold of the protein, and has a CLASP score of zero. All matches below a user defined threshold score (S_thresh_) are discarded. The index for the degree of specificity should encapsulate the number of scaffolds that are similar to the original active site, as well as the quality of the matches which are reflected by the CLASP scores (Equation 4). Furthermore, we ignore scaffolds which do not include at least one of the known active site residues. Such a moonlighting activity, even if true, is not of interest in the current work.

The BRASS methodology can be extended to incorporate stereochemically equivalent residues in the match. Each position of the motif can have a set of amino acids specified to allow for stereochemically equivalent matches at that particular position (Equation 5). This introduces an additional constraint while matching each residue, ensuring that the amino acid type of r_i_ (Equation 2) belongs to GROUP_i_ (Equation 5).
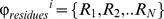
(1)

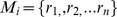
(2)

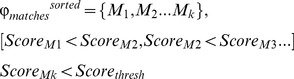
(3)

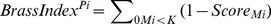
(4)

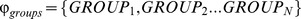
(5)To summarize, the active site signature for each protein is used to query itself, generating a list of scaffolds with an associated CLASP score. Lower CLASP scores denote better congruence, and we discard all matches whose scores are more than a user defined threshold. The BrassIndex for the enzymatic function of a protein is now defined by the number of scaffolds that can serve as ‘replicas’ of the original scaffold. The BrassIndex is normalized such that the protein with the broadest specificity has BrassIndex of 1.

An inherent limitation of CLASP, and typical of other methods that use RMSD, is its inability to distinguish between mirror images. Thus, permutations of the original active site are also excluded. We compute the BrassIndex for a non-homologous set of proteins with known active site and structure extracted from the CSA database [24]. The source code has been made available at www.sanchak.com/brass.html.

### 3. Dataset Selection

CSA provides catalytic residue annotation for enzymes in the PDB and is available online [24]. The database consists of an original hand-annotated set extracted from the primary literature and a homologous set inferred by PSI-BLAST [74]. The motifs picked were those that were extracted from the literature, had either 3, 4 or 5 residues and were all confined to one polypeptide. The extended set of proteins now has 420 proteins. Some proteins were excluded since they failed the electrostatic analysis. Table S3 shows the proteins in our test set.

## Supporting Information

Table S1 **BRASS results for Cytochrome P450 3A4 (PDBid:3UA1): A flexible loop (residues 211–218) is proposed to be the reason for the broad substrate specificity** [**63**] **.** BRASS identifies four possibilities for the torsional position of Phe215, which is shown to have the maximum shift upon substrate binding. The core catalytic residues are - Thr309, Glu308, Phe435 and Cys442.
(PDF)

Table S2 **Potential and spatial congruence of the active site residues in serine proteases: Chymotrypsin and subtilisin are a classical example of convergent evolution where the catalytic Ser-His-Asp triad shows virtually similar geometry in the structurally different proteins.** D = Pairwise distance in Å. PD = Pairwise potential difference. See Methods section for units of potential.
(PDF)

Table S3 **Set of non-homologous proteins with known active site residues: The proteins are ordered in an ascending order with respect to the BrassIndex.**
(PDF)
