# Supplementary material for: A Measure of the Broad Substrate Specificity of Enzymes Based on ‘Duplicate’ Catalytic Residues
Source: PLoS One. 2012 Nov 16;7(11):e49313. doi: 10.1371/journal.pone.0049313 (PMC3500292; doi:10.1371/journal.pone.0049313)
Supplement: Table S1 — BRASS results for Cytochrome P450 3A4 (PDBid:3UA1): A flexible loop (residues 211–218) is proposed to be the reason for the broad substrate specificity [63] . BRASS identifies four possibilities for the torsional position of Phe215, which is shown to have the maximum shift upon substrate binding. The core catalytic residues are - Thr309, Glu308, Phe435 and Cys442. (PDF) [file pone.0049313.s001.pdf]

Supplementary Table. 1: **BRASS results for Cytochrome P450 3A4 (PDBid:3UA1)**: A flexilble loop (residues 211-218) is proposed to be the reason for the broad substrate specificity [63]. BRASS identifies four possibilities for the torsional position of Phe215, which is shown to have the maximum shift upon substrate binding. The core catalytic residues are - Thr309, Glu308, Phe435 and Cys442.

PHE215CZ	D	8.1	8.7	7.6	13.9	14.7	14.8	12.3	5.9	19.2	16.0
	PD	231.2	-163.2	-151.1	-144.9	-394.4	-382.3	-376.2	12.1	18.3	6.2
PHE213CZ	D	8.1	8.7	7.6	13.4	14.7	14.8	12.5	5.9	20.7	16.8
	PD	231.2	-163.2	-151.1	-118.0	-394.4	-382.3	-349.2	12.1	45.2	33.1
PHE241CZ	D	8.1	8.7	7.6	13.2	14.7	14.8	14.6	5.9	19.9	15.2
	PD	231.2	-163.2	-151.1	-159.6	-394.4	-382.3	-390.9	12.1	3.6	-8.5
PHE108CZ	D	8.1	8.7	7.6	15.3	14.7	14.8	14.8	5.9	21.1	16.9
	PD	231.2	-163.2	-151.1	-154.5	-394.4	-382.3	-385.8	12.1	8.7	-3.4
