# Supplementary material for: A Measure of the Broad Substrate Specificity of Enzymes Based on ‘Duplicate’ Catalytic Residues
Source: PLoS One. 2012 Nov 16;7(11):e49313. doi: 10.1371/journal.pone.0049313 (PMC3500292; doi:10.1371/journal.pone.0049313)
Supplement: Table S2 — Potential and spatial congruence of the active site residues in serine proteases: Chymotrypsin and subtilisin are a classical example of convergent evolution where the catalytic Ser-His-Asp triad shows virtually similar geometry in the structurally different proteins. D = Pairwise distance in Å. PD = Pairwise potential difference. See Methods section for units of potential. (PDF) [file pone.0049313.s002.pdf]

Supplementary Table. 2: **Potential and spatial congruence of the active site residues in serine proteases**: Chymotrypsin and subtilisin are a classical example of convergent evolution where the catalytic Ser-His-Asp triad shows virtually similar geometry in the structurally different proteins. D = Pairwise distance in Å. PD = Pairwise potential difference. See Methods section for units of potential.

PDB	Active site atoms(a,b,c)		ab	ac	bc
1A0J	ASP102OD1,SER195OG,HIS57NE2,	D	7.8	5.6	3.3
	chymotrypsin	PD	-144.1	-39.2	104.8
1BIT	ASP102OD1,SER195OG,HIS57NE2,	D	8.0	5.6	3.3
	chymotrypsin	PD	-120.5	21.4	142.0
1AF4	ASP32OD1,SER221OG,HIS64NE2,	D	7.5	4.7	3.3
	subtilisin	PD	-139.4	45.3	184.7
1BH6	ASP32OD1,SER221OG,HIS64NE2,	D	6.6	4.9	2.8
	subtilisin	PD	-90.4	32.0	122.4
