# Supplementary material for: A Measure of the Broad Substrate Specificity of Enzymes Based on ‘Duplicate’ Catalytic Residues
Source: PLoS One. 2012 Nov 16;7(11):e49313. doi: 10.1371/journal.pone.0049313 (PMC3500292; doi:10.1371/journal.pone.0049313)
Supplement: Table S3 — Set of non-homologous proteins with known active site residues: The proteins are ordered in an ascending order with respect to the BrassIndex. (PDF) [file pone.0049313.s003.pdf]

Supplementary Table. 3: Set of non-homologous proteins with known active site residues: The proteins are ordered in an ascending order with respect to the BrassIndex.

PDB	Sequence length	Native function
1BWP	233	PLATELET-ACTIVATING FACTOR ACETYLHYDROLASE
1AKO	268	EXONUCLEASE III
1L1L	739	RIBONUCLEOSIDE TRIPHOSPHATE REDUCTASE
2DLN	306	D-ALANINE–D-ALANINE LIGASE
1NWW	149	Limonene-1,2-epoxide hydrolase
1EI5	520	D-AMINOPEPTIDASE
1NBA	264	N-CARBAMOYLSARCOSINE AMIDOHYDROLASE
1EC9	446	GLUCARATE DEHYDRATASE
1STD	172	SCYTALONE DEHYDRATASE
1EHI	377	D-ALANINE:D-LACTATE LIGASE
1E0C	271	SULFURTRANSFERASE
1AGY	200	CUTINASE
1G6T	427	EPSP SYNTHASE
1AUO	218	CARBOXYLESTERASE
1FCQ	350	HYALURONOGLUCOSAMINIDASE
1BS9	207	ACETYL XYLAN ESTERASE
1HDH	536	ARYLSULFATASE
1G99	408	ACETATE KINASE
1A8S	273	CHLOROPEROXIDASE F
1A0J	223	TRYPSIN
1A8H	500	METHIONYL-TRNA SYNTHETASE
1APX	249	CYTOSOLIC ASCORBATE PEROXIDASE
1ASY	490	ASPARTYL-tRNA SYNTHETASE
1B2M	104	RIBONUCLEASE T1
1B57	358	PROTEIN (FRUCTOSE-BISPHOSPHATE ALDOLASE II)
1B5D	246	PROTEIN (DEOXYCYTIDYLATE HYDROXYMETHYLASE)
1B6G	310	HALOALKANE DEHALOGENASE
1B6T	159	PROTEIN (PHOSPHOPANTETHEINE ADENYLYLTRANSFERA
1B8G	429	PROTEIN (1-AMINOCYCLOPROPANE-1-CARBOXYLATE SY
1BBS	340	RENIN
1BHG	613	BETA-GLUCURONIDASE
1BJO	360	PHOSPHOSERINE AMINOTRANSFERASE
1BMT	246	METHIONINE SYNTHASE
1BRM	367	ASPARTATE-SEMIALDEHYDE DEHYDROGENASE
1C82	731	HYALURONATE LYASE
1CDE	212	PHOSPHORIBOSYL-GLYCINAMIDE FORMYLTRANSFERASE
1CNS	243	CHITINASE
1COY	507	CHOLESTEROL OXIDASE
1D7R	433	PROTEIN (2,2-DIALKYLGLYCINE DECARBOXYLASE (PY
1DAA	282	D-AMINO ACID AMINOTRANSFERASE
1DBT	239	OROTIDINE 5'-PHOSPHATE DECARBOXYLASE
1DHP	292	DIHYDRODIPICOLINATE SYNTHASE
1DHR	241	DIHYDROPTERIDINE REDUCTASE
1DJL	207	TRANSHYDROGENASE DIII
1DNP	471	DNA PHOTOLYASE

Continued on Next Page…

Supplementary Table. 3 – Continued

PDB	Sequence length	Native function
1DPT	117	D-DOPACHROME TAUTOMERASE
1E2T	284	N-HYDROXYARYLAMINE O-ACETYLTRANSFERASE
1EF8	261	METHYLMALONYL COA DECARBOXYLASE
1EUY	548	GLUTAMINYL-TRNA SYNTHETASE
1F8M	429	ISOCITRATE LYASE
1FNB	314	FERREDOXIN-NADP+ REDUCTASE
1FOB	334	BETA-1,4-GALACTANASE
1FOH	664	PHENOL HYDROXYLASE
1GAL	583	GLUCOSE OXIDASE
1GEQ	248	TRYPTOPHAN SYNTHASE ALPHA-SUBUNIT
1GIM	431	ADENYLOSUCCINATE SYNTHETASE
1GUM	222	PROTEIN (GLUTATHIONE TRANSFERASE A4-4)
1HRK	359	FERROCHELATASE
1IG8	486	hexokinase PII
1IPH	753	CATALASE HPII
1K4T	592	DNA topoisomerase I
1MBB	342	URIDINE DIPHOSPHO-N-ACETYLENOLPYRUVYLGLUCOSAM
1NMW	114	Peptidyl-prolyl cis-trans isomerase NIMA-inte
1ONR	316	TRANSALDOLASE B
1ORD	730	ORNITHINE DECARBOXYLASE
1PAD	212	PAPAIN
1PO5	476	Cytochrome P450 2B4
1QFE	252	PROTEIN (3-DEHYDROQUINATE DEHYDRATASE)
1QFM	710	PROTEIN (PROLYL OLIGOPEPTIDASE)
1QX3	257	Apopain
1RHS	296	SULFUR-SUBSTITUTED RHODANESE
1SCA	274	SUBTILISIN CARLSBERG
1VCM	550	CTP synthetase
1YSC	421	SERINE CARBOXYPEPTIDASE
1ZRZ	364	Protein kinase C, iota
2ACE	537	ACETYLCHOLINESTERASE
2AMG	418	1,4-ALPHA-D-GLUCAN MALTOTETRAHYDROLASE
2GSA	432	GLUTAMATE SEMIALDEHYDE AMINOTRANSFERASE
2ISD	624	PHOSPHOINOSITIDE-SPECIFIC PHOSPHOLIPASE C, IS
2LPR	198	ALPHA-LYTIC PROTEASE
2OAT	439	ORNITHINE AMINOTRANSFERASE
2TMD	729	TRIMETHYLAMINE DEHYDROGENASE
3CLA	213	TYPE III CHLORAMPHENICOL ACETYLTRANSFERASE
5FIT	147	FRAGILE HISTIDINE TRIAD PROTEIN
7ODC	424	PROTEIN (ORNITHINE DECARBOXYLASE)
8PCH	220	CATHEPSIN H
1A26	361	POLY (ADP-RIBOSE) POLYMERASE
1A95	152	XANTHINE-GUANINE PHOSPHORIBOSYLTRANSFERASE
1AFR	345	DELTA9 STEAROYL-ACYL CARRIER PROTEIN DESATURA
1AFW	393	3-KETOACETYL-COA THIOLASE
1AQ0	306	1,3-1,4-BETA-GLUCANASE
1AUI	521	SERINE/THREONINE PHOSPHATASE 2B

Continued on Next Page…

Supplementary Table. 3 – Continued

PDB	Sequence length	Native function
1B66	140	6-PYRUVOYL TETRAHYDROPTERIN SYNTHASE
1BRW	433	PROTEIN (PYRIMIDINE NUCLEOSIDE PHOSPHORYLASE)
1BS0	384	PROTEIN (8-AMINO-7-OXONANOATE SYNTHASE)
1BSJ	168	PROTEIN (PEPTIDE DEFORMYLASE)
1BTL	263	BETA-LACTAMASE TEM1
1BVZ	585	PROTEIN (ALPHA-AMYLASE II)
1BWD	348	PROTEIN (INOSAMINE-PHOSPHATE AMIDINOTRANSFERA
1BZC	321	PROTEIN (PROTEIN-TYROSINE-PHOSPHATASE)
1C3J	351	BETA-GLUCOSYLTRANSFERASE
1C4Z	358	UBIQUITIN-PROTEIN LIGASE E3A
1C9U	454	SOLUBLE QUINOPROTEIN GLUCOSE DEHYDROGENASE
1CD5	266	PROTEIN (GLUCOSAMINE 6-PHOSPHATE DEAMINASE)
1CEL	434	1,4-BETA-D-GLUCAN CELLOBIOHYDROLASE I
1CGK	389	PROTEIN (CHALCONE SYNTHASE)
1CMX	235	PROTEIN (UBIQUITIN YUH1-UBAL)
1CV2	296	HALOALKANE DEHALOGENASE
1CVR	435	GINGIPAIN R
1D3G	367	DIHYDROOROTATE DEHYDROGENASE
1D4C	572	FLAVOCYTOCHROME C FUMARATE REDUCTASE
1DAE	224	DETHIOBIOTIN SYNTHETASE
1DB3	372	GDP-MANNOSE 4,6-DEHYDRATASE
1DGS	667	DNA LIGASE
1DI1	300	ARISTOLOCHENE SYNTHASE
1DL2	511	CLASS I ALPHA-1,2-MANNOSIDASE
1DWO	262	HYDROXYNITRILE LYASE
1E7Q	321	GDP-FUCOSE SYNTHETASE
1EH6	207	O6-ALKYLGUANINE-DNA ALKYLTRANSFERASE
1EYP	222	CHALCONE-FLAVONONE ISOMERASE 1
1F8X	157	NUCLEOSIDE 2-DEOXYRIBOSYLTRANSFERASE
1FUI	591	L-FUCOSE ISOMERASE
1G0D	695	PROTEIN-GLUTAMINE GAMMA-GLUTAMYLTRANSFERASE
1GCB	454	GAL6 HG (EMTS) DERIVATIVE
1GLO	217	CATHEPSIN S
1GPR	162	GLUCOSE PERMEASE
1GQ8	319	PECTINESTERASE
1ITX	419	Glycosyl Hydrolase
1J49	333	D-LACTATE DEHYDROGENASE
1JM6	407	Pyruvate dehydrogenase kinase, isozyme 2
1JXA	608	glucosamine 6-phosphate synthase
1KAE	434	Histidinol dehydrogenase
1L7D	384	nicotinamide nucleotide Transhydrogenase, sub
1MEK	120	PROTEIN DISULFIDE ISOMERASE
1MPP	361	PEPSIN
1NLN	204	Adenain
1OG1	226	T-CELL ECTO-ADP-RIBOSYLTRANSFERASE 2
1PA9	284	Protein-tyrosine phosphatase yopH
1PTD	298	PHOSPHATIDYLINOSITOL-SPECIFIC PHOSPHOLIPASE C

Continued on Next Page…

Supplementary Table. 3 – Continued

PDB	Sequence length	Native function
1Q6L	216	3-keto-L-gulonate 6-phosphate decarboxylase
1QHO	686	ALPHA-AMYLASE
1QK2	363	CELLOBIOHYDROLASE CEL6A (FORMERLY CALLED CBH
1QRR	394	sulfolipid biosynthesis (SQD1) PROTEIN
1RPX	230	PROTEIN (RIBULOSE-PHOSPHATE 3-EPIMERASE)
1SME	329	PLASMEPSIN II
1UCH	230	UBIQUITIN C-TERMINAL HYDROLASE UCH-L3
1VAS	137	PROTEIN (T4 ENDONUCLEASE V (E.C.3.1.25.1))
1XYZ	347	1,4-BETA-D-XYLAN-XYLANOHYDROLASE
1YBV	283	TRIHYDROXYNAPHTHALENE REDUCTASE
1Z9H	290	membrane-associated prostaglandin E synthase-
1ZE1	309	tRNA pseudouridine synthase B
2GVW	314	Phosphotriesterase
2NAC	393	NAD-DEPENDENT FORMATE DEHYDROGENASE
2PFL	759	PROTEIN (PYRUVATE FORMATE-LYASE)
2PGD	482	6-PHOSPHOGLUCONATE DEHYDROGENASE
3CSM	256	CHORISMATE MUTASE
3PVA	335	PROTEIN (PENICILLIN V ACYLASE)
1UF7	303	N-carbamyl-D-amino acid amidohydrolase
1A4S	503	BETAINE ALDEHYDE DEHYDROGENASE
1NLU	370	SEDOLISIN
1DE6	426	L-RHAMNOSE ISOMERASE
5ENL	436	ENOLASE
1AZW	313	PROLINE IMINOPEPTIDASE
1CA3	260	CARBONIC ANHYDRASE II
1RK2	309	RIBOKINASE
1EQ2	310	ADP-L-GLYCERO-D-MANNOHEPTOSE 6-EPIMERASE
1PFQ	731	Dipeptidyl peptidase IV soluble form
1NHX	625	PHOSPHOENOLPYRUVATE CARBOXYKINASE, CYTOSOLIC
1QRZ	246	PLASMINOGEN
1UOK	558	OLIGO-1,6-GLUCOSIDASE
1I78	297	PROTEASE VII
1UK7	282	2-hydroxy-6-oxo-7-methylocta-2,4-dienoate hyd
1K82	268	formamidopyrimidine-DNA glycosylase
1OYA	400	OLD YELLOW ENZYME
1DII	521	P-CRESOL METHYLHYDROXYLASE
1N2T	386	L-cysteine/cystine lyase C-DES
1KEZ	300	ERYTHRONOLIDE SYNTHASE
132L	129	HEN EGG WHITE LYSOZYME
1WNW	215	Heme oxygenase
1XS1	193	Deoxycytidine triphosphate deaminase
1EU1	780	DIMETHYL SULFOXIDE REDUCTASE
1PXV	183	cysteine protease
1POW	585	PYRUVATE OXIDASE
1SSX	198	Alpha-lytic protease
1ECL	597	ESCHERICHIA COLI TOPOISOMERASE I
1D4A	273	QUINONE REDUCTASE

Continued on Next Page…

Supplementary Table. 3 – Continued

PDB	Sequence length	Native function
2ADM	421	ADENINE-N6-DNA-METHYLTRANSFERASE TAQI
1C4X	285	PROTEIN (2-HYDROXY-6-OXO-6-PHENYLHEXA-2,4-DIE
1CW0	155	PROTEIN (DNA MISMATCH ENDONUCLEASE)
1A79	171	TRNA ENDONUCLEASE
1GOG	639	GALACTOSE OXIDASE
2F9R	285	Sphingomyelinase D 1
1BWZ	274	PROTEIN (DIAMINOPIMELATE EPIMERASE)
1BIX	287	AP ENDONUCLEASE 1
2LIP	320	LIPASE
1MHT	327	PROTEIN (HHAI METHYLTRANSFERASE)
1DJ1	291	CYTOCHROME C PEROXIDASE
1PYM	295	PROTEIN (PHOSPHOENOLPYRUVATE MUTASE)
1T7D	250	SIGNAL PEPTIDASE I
1M9C	165	Cyclophilin A
1INP	400	INOSITOL POLYPHOSPHATE 1-PHOSPHATASE
2BIF	469	PROTEIN (6-PHOSPHOFRUCTO-2-KINASE/FRUCTOSE-2,
1EEJ	216	THIOL:DISULFIDE INTERCHANGE PROTEIN
1GA8	311	GALACTOSYL TRANSFERASE LGTC
1EUU	605	SIALIDASE
1CG6	283	PROTEIN (5'-DEOXY-5'-METHYLTHIOADENOSINE PHOS
1FR8	288	BETA 1,4 GALACTOSYLTRANSFERASE
1PJB	361	L-ALANINE DEHYDROGENASE
1EHY	294	PROTEIN (SOLUBLE EPOXIDE HYDROLASE)
1DE3	150	RIBONUCLEASE ALPHA-SARCIN
1CZF	362	POLYGALACTURONASE II
1RHC	330	F420-dependent alcohol dehydrogenase
1TEH	373	HUMAN CHICHI ALCOHOL DEHYDROGENASE
206L	164	LYSOZYME
2TOH	343	TYROSINE 3-MONOOXYGENASE
1JOF	365	CARBOXY-CIS,CIS-MUCONATE CYCLASE
1OJ4	283	4-DIPHOSPHOCYTIDYL-2-C-METHYL-D-ERYTHRITOL KI
1NBF	353	Ubiquitin carboxyl-terminal hydrolase 7
1MPX	615	alpha-amino acid ester hydrolase
1DIZ	282	3-METHYLADENINE DNA GLYCOSYLASE II
1F7U	607	ARGINYL-TRNA SYNTHETASE
1H7O	341	5-AMINOLAEVULINIC ACID DEHYDRATASE
1DUP	152	DEOXYURIDINE 5'-TRIPHOSPHATE NUCLEOTIDOHYDROL
1BP2	123	PHOSPHOLIPASE A2
1J7G	144	D-tyrosyl-tRNA(Tyr) deacylase
2PLC	274	PHOSPHATIDYLINOSITOL-SPECIFIC PHOSPHOLIPASE C
1M0T	491	glutathione synthetase
1J70	514	ATP SULPHURYLASE
1QZ9	416	KYNURENINASE
1TRK	680	TRANSKETOLASE
1Q91	197	5(3)-deoxyribonucleotidase
1VAO	560	VANILLYL-ALCOHOL OXIDASE
1RPT	342	PROSTATIC ACID PHOSPHATASE

Continued on Next Page...

Supplementary Table. 3 – Continued

PDB	Sequence length	Native function
1WD8	670	Protein-arginine deiminase type IV
1IR3	306	INSULIN RECEPTOR
1AJ0	282	DIHYDROPTEROATE SYNTHASE
1ILE	821	ISOLEUCYL-TRNA SYNTHETASE
1AM2	199	MXE GYRA INTEIN
1HPL	449	LIPASE
1TZ3	339	putative sugar kinase
1P7M	187	DNA-3-methyladenine glycosylase I
1TDE	316	THIOREDOXIN REDUCTASE
1QSG	265	ENOYL-[ACYL-CARRIER-PROTEIN] REDUCTASE
1AGM	470	GLUCOAMYLASE-471
1V0Y	506	PHOSPHOLIPASE D
2CND	270	NADH-DEPENDENT NITRATE REDUCTASE
2DW7	389	Bll6730 protein
1ZNB	232	METALLO-BETA-LACTAMASE
1D5R	324	PHOSPHOINOSITIDE PHOSPHOTASE PTEN
2F61	497	Acid beta-glucosidase
1NAA	541	Cellobiose dehydrogenase
1A69	238	PURINE NUCLEOSIDE PHOSPHORYLASE
1ECF	504	GLUTAMINE PHOSPHORIBOSYLPYROPHOSPHATE AMIDOTR
1AMY	403	1,4-ALPHA-D-GLUCAN GLUCANOHYDROLASE
2DHN	121	7,8-DIHYDRONEOPTERIN ALDOLASE
1GOX	370	(S)-2-HYDROXY-ACID OXIDASE, PEROXISOMAL
1EAG	342	ASPARTIC PROTEINASE (SAP2 GENE PRODUCT)
1B2R	304	PROTEIN (FERREDOXIN-NADP+ REDUCTASE)
1JDW	423	L-ARGININE:GLYCINE AMIDINOTRANSFERASE
1AJ8	371	CITRATE SYNTHASE
1GPA	842	GLYCOGEN PHOSPHORYLASE A
1LDM	329	M4 LACTATE DEHYDROGENASE
1MJ9	278	ESA1 PROTEIN
1D8C	723	MALATE SYNTHASE G
1DPG	485	GLUCOSE 6-PHOSPHATE DEHYDROGENASE
1HXQ	348	HEXOSE-1-PHOSPHATE URIDYLYLTRANSFERASE
1MUC	373	MUCONATE LACTONIZING ENZYME
2ACU	315	ALDOSE REDUCTASE
2QMO	220	Dethiobiotin synthetase
1OBA	339	LYSOZYME
2ENG	210	ENDOGLUCANASE V
3R1R	761	RIBONUCLEOTIDE REDUCTASE R1 PROTEIN
1BF2	750	ISOAMYLASE
1RTU	114	RIBONUCLEASE U2
2A0N	265	Imidazole glycerol phosphate synthase subunit
1FY2	229	ASPARTYL DIPEPTIDASE
1CWY	500	AMYLOMALTASE
1D1Q	161	TYROSINE PHOSPHATASE (E.C.3.1.3.48)
1GRC	212	GLYCINAMIDE RIBONUCLEOTIDE TRANSFORMYLASE
2HDH	293	L-3-HYDROXYACYL COA DEHYDROGENASE

Continued on Next Page. . .

Supplementary Table. 3 – Continued

PDB	Sequence length	Native function
1FCB	511	FLAVOCYTOCHROME B2
1KQC	217	OXYGEN-INSENSITIVE NAD(P)H NITROREDUCTASE
1LZ1	130	HUMAN LYSOZYME
1CTN	540	CHITINASE A
1B73	254	GLUTAMATE RACEMASE
1PII	452	N-(5'PHOSPHORIBOSYL)ANTHRANILATE ISOMERASE
1PS1	337	PENTALENENE SYNTHASE
1AL6	437	CITRATE SYNTHASE
1GDH	320	D-GLYCERATE DEHYDROGENASE
1R6W	322	o-Succinylbenzoate Synthase
1OTG	125	5-CARBOXYMETHYL-2-HYDROXYMUCONATE ISOMERASE
1WKB	810	Leucyl-tRNA synthetase
2NMT	422	MYRISTOYL-COA:PROTEIN N-MYRISTOYLTRANSFERASE
1VNC	609	VANADIUM-CONTAINING CHLOROPEROXIDASE
1KNP	540	L-aspartate oxidase
1PFK	320	PHOSPHOFRUCTOKINASE
1CHM	401	CREATINE AMIDINOHYDROLASE
2AYH	214	1,3-1,4-BETA-D-GLUCAN 4-GLUCANOHYDROLASE
1LUC	355	BACTERIAL LUCIFERASE
1YLU	217	Oxygen-insensitive NAD(P)H nitroreductase
1AUK	489	ARYLSULFATASE A
2THI	379	THIAMINASE I
1DNK	260	PROTEIN (DEOXYRIBONUCLEASE I (DNASE I) (E.C.3
2ESD	475	NADP-dependent glyceraldehyde-3-phosphate deh
1L9X	315	gamma-glutamyl hydrolase
1MDR	359	MANDELATE RACEMASE
5RSA	124	RIBONUCLEASE A
1SMN	245	EXTRACELLULAR ENDONUCLEASE
1OAS	322	O-ACETYLSERINE SULFHYDRYLASE
1HY3	294	ESTROGEN SULFOTRANSFERASE
1DDJ	247	PLASMINOGEN
1FVA	217	PEPTIDE METHIONINE SULFOXIDE REDUCTASE
1QHF	240	PROTEIN (PHOSPHOGLYCERATE MUTASE)
1E19	314	CARBAMATE KINASE-LIKE CARBAMOYLPHOSPHATE SYNT
1GSA	316	GLUTATHIONE SYNTHETASE
1FO6	304	N-CARBAMoYL-D-AMINO-ACID AMIDOHYDROLASE
1MLA	309	MALONYL-COENZYME A ACYL CARRIER PROTEIN TRANS
1MOQ	368	GLUCOSAMINE 6-PHOSPHATE SYNTHASE
1BQC	302	PROTEIN (BETA-MANNANASE)
5EAT	548	5-EPI-ARISTOLOCHENE SYNTHASE
1QBA	858	CHITOBIASE
1PNT	157	ACID PHOSPHATASE
1A4L	349	ADENOSINE DEAMINASE
1Y9M	518	exo-inulinase
1D2R	326	PROTEIN (TRYPTOPHANYL TRNA SYNTHETASE)
1BU7	455	PROTEIN (CYTOCHROME P450)
1KAS	412	BETA-KETOACYL ACP SYNTHASE II

Continued on Next Page…

Supplementary Table. 3 – Continued

PDB	Sequence length	Native function
1FJO	316	THERMOLYSIN
2TPS	227	PROTEIN (THIAMIN PHOSPHATE SYNTHASE)
2EQL	129	HORSE MILK LYSOZYME
1I9A	182	ISOPENTENYL-DIPHOSPHATE DELTA-ISOMERASE
1BOO	323	PROTEIN (N-4 CYTOSINE-SPECIFIC METHYLTRANSFER
1CBG	490	CYANOGENIC BETA-GLUCOSIDASE
1XQD	403	CYTOCHROME P450 55A1
1MAS	314	INOSINE-URIDINE NUCLEOSIDE N-RIBOHYDROLASE
1DOD	394	P-HYDROXYBENZOATE HYDROXYLASE
1CDG	686	CYCLODEXTRIN GLYCOSYL-TRANSFERASE
1FC4	401	2-AMINO-3-KETOBUTYRATE CONENZYME A LIGASE
2BHG	209	FOOT-AND-MOUTH DISEASE VIRUS 3C PROTEASE
1JS4	605	ENDO/EXOCELLULASE E4
1MYR	501	MYROSINASE
1X7D	350	ornithine cyclodeaminase
1DIO	554	PROTEIN (DIOL DEHYDRATASE)
1UAE	419	UDP-N-ACETYLGLUCOSAMINE ENOLPYRUVYL TRANSFERA
1S95	333	Serine/threonine protein phosphatase 5
1ALD	363	ALDOLASE A
1GXS	158	P-(S)-HYDROXYMANDELONITRILE LYASE CHAIN B
1AM5	324	PEPSIN
1BG6	359	N-(1-D-CARBOXYLETHYL)-L-NORVALINE DEHYDROGENA
1L1D	152	peptide methionine sulfoxide reductase
1F6D	376	UDP-N-ACETYLGLUCOSAMINE 2-EPIMERASE
1FQ0	213	KDPG ALDOLASE
1BE1	137	GLUTAMATE MUTASE
1BG0	356	ARGININE KINASE
1CQQ	180	TYPE 2 RHINOVIRUS 3C PROTEASE
1NKK	256	Capsid protein P40
1MPY	307	CATECHOL 2,3-DIOXYGENASE
2PTH	193	PEPTIDYL-TRNA HYDROLASE
1THT	305	THIOESTERASE
1PJH	280	enoyl-CoA isomerase; Eci1p
1CQG	105	THIOREDOXIN
1PGS	314	PEPTIDE-N(4)-(N-ACETYL-BETA-D-GLUCOSAMINYL)AS
2AAT	396	ASPARTATE AMINOTRANSFERASE
1AT1	310	ASPARTATE CARBAMOYLTRANSFERASE, CATALYTIC CHA
5CPA	307	CARBOXYPEPTIDASE A
1SLL	679	SIALIDASE L
1AQ2	540	PHOSPHOENOLPYRUVATE CARBOXYKINASE
1A7U	277	CHLOROPEROXIDASE T
2CPU	496	ALPHA-AMYLASE
1LAM	484	LEUCINE AMINOPEPTIDASE
1RNE	340	RENIN
1EXP	312	BETA-1,4-D-GLYCANASE CEX-CD
1BGL	1023	BETA-GALACTOSIDASE
1JKM	361	BREFELDIN A ESTERASE

Continued on Next Page...

Supplementary Table. 3 – Continued

PDB	Sequence length	Native function
1HKA	158	6-HYDROXYMETHYL-7,8-DIHYDROPTERIN PYROPHOSPHO
1QAZ	351	PROTEIN (ALGINATE LYASE A1-III)
1QE3	489	PARA-NITROBENZYL ESTERASE
1CA1	370	ALPHA-TOXIN
1D8H	311	mRNA TRIPHOSPHATASE CET1
1IEC	256	CAPSID PROTEIN P40: ASSEMBLIN PROTEASE
1RBL	467	RIBULOSE 1,5 BISPHOSPHATE CARBOXYLASE/OXYGENA
1AST	200	ASTACIN
1M53	570	Isomaltulose Synthase
1V25	541	long-chain-fatty-acid-CoA synthetase
1DO8	564	MALIC ENZYME
1THG	544	LIPASE
1A16	440	AMINOPEPTIDASE P
1V0E	666	ENDO-ALPHA-SIALIDASE
5COX	587	CYCLOOXYGENASE-2
1OPM	310	PROTEIN (PEPTIDYLGLYCINE ALPHA-HYDROXYLATING
1QOL	173	PROTEASE (NONSTRUCTURAL PROTEIN P20A)
2HGS	474	PROTEIN (GLUTATHIONE SYNTHETASE)
1BOL	222	PROTEIN (RIBONUCLEASE RH)
1SNZ	344	aldose 1-epimerase
1ARZ	273	DIHYDRODIPICOLINATE REDUCTASE
1C7H	131	DELTA-5-3-KETOSTEROID ISOMERASE
1OYG	447	levansucrase
1L6P	125	Thiol:disulfide interchange protein dsbD
1VZZ	131	STEROID DELTA-ISOMERASE
1CMS	323	PROCHYMOSIN A/B PRECURSOR
1B9H	388	PROTEIN (3-AMINO-5-HYDROXYBENZOIC ACID SYNTHA
1E2A	105	ENZYME IIA
2YPN	313	PROTEIN (HYDROXYMETHYLBILANE SYNTHASE)
1D2H	292	GLYCINE N-METHYLTRANSFERASE
1LI5	461	CYSTEINYL-TRNA SYNTHETASE
1TDJ	514	BIOSYNTHETIC THREONINE DEAMINASE
1OXA	403	CYTOCHROME P450 ERYF
1DUB	261	2-ENOYL-COA HYDRATASE
1LJL	131	arsenate reductase
1R76	408	pectate lyase
1PWV	776	Lethal factor
1DCI	275	DIENOYL-COA ISOMERASE
1UAS	362	alpha-galactosidase
1EH5	279	PALMITOYL PROTEIN THIOESTERASE 1
1HZD	272	AU-BINDING PROTEIN/ENOYL-COA HYDRATASE
1P1X	260	Deoxyribose-phosphate aldolase
2JXR	329	PROTEINASE A
1QGX	357	3',5'-ADENOSINE BISPHOSPHATASE
1AKM	333	ORNITHINE TRANSCARBAMYLASE
1OH9	258	ACETYLGLUTAMATE KINASE
1QAM	244	ERMC' METHYLTRANSFERASE
